# The Cutaneous Ciliated Cyst in Young Male: The Possibility of Ciliated Cutaneous Eccrine Cyst

**DOI:** 10.1155/2015/589831

**Published:** 2015-09-30

**Authors:** Youngjoon Kim, Hyunjung Kim

**Affiliations:** ^1^Department of Plastic and Reconstructive Surgery, Sanggye Paik Hospital, Inje University College of Medicine, 1342 Dongil-ro, Nowon-gu, Seoul 139-707, Republic of Korea; ^2^Department of Pathology, Sanggye Paik Hospital, Inje University College of Medicine, 1342 Dongil-ro, Nowon-gu, Seoul 139-707, Republic of Korea

## Abstract

Cutaneous ciliated cyst was described as a painless cyst occurring on the lower limbs of women between the ages of 15 and 30 years. The cysts are typically lined by ciliated cuboidal to columnar epithelium with pseudostratified areas and focal squamous metaplasia is occasionally present. Immunohistochemical studies have demonstrated that the cysts are PR and ER positive, similar to the epithelia of the fallopian tubes. However, outliers of cutaneous ciliated cysts, including those in male patients and in unexpected locations such as the scalp, finger, and scapular area, have been reported. Thus, some hypotheses have been proposed including the Mullerian heterotopias, ciliated metaplasia of eccrine sweat glands, and embryonic remnants of the cloacal membrane. We report a rare case of cutaneous ciliated cyst on the left shoulder of a 7-year-old boy and this is the eighth case of cutaneous ciliated cyst in male patients. Moreover, through reviewing the articles, we try to propose the classification of the cutaneous ciliated cysts into the cutaneous Mullerian cysts and the ciliated cutaneous eccrine cysts.

## 1. Introduction

Cutaneous ciliated cysts are rare benign lesions and are typically lined by a cuboidal to columnar ciliated epithelium, with some areas of pseudostratified ciliated epithelium [1]. Cutaneous ciliated cysts were originally described as a painless cyst occurring on the lower limbs of women between the ages of 15 and 30 years [2]. Because of the similarities between the epithelium of the fallopian tubes and cutaneous ciliated cysts, Mullerian heterotopias have been proposed as a possible pathogenesis [1, 2]. Moreover, nuclear positivity for sex steroid receptors, such as the estrogen receptor (ER) and progesterone receptor (PR), has been observed in immunohistochemical staining, which is suggestive of Mullerian heterotopia [3, 4]. However, in recent years, there have been other hypotheses such as eccrine origin [5] and cloacal membrane origin [6]. The eccrine metaplasia hypothesis was proposed following the identification of morphologically similar cysts in male patients [5]. Some authors have suggested that it was originated from an embryonic remnant of the cloacal membrane because of the observed perineal locations [6, 7].

We report a case of a cutaneous ciliated cyst on the shoulder of a 7-year-old boy and propose the classification based on a review of articles describing cutaneous ciliated cysts.

## 2. Case Presentation

**Figure 1:**
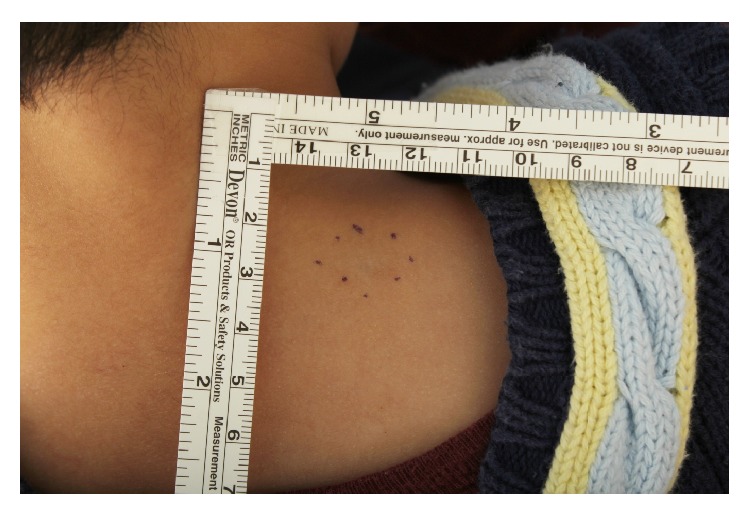
Clinical photography of a lesion shows a solitary cyst in the left posterior neck region, 1 cm diameter.

**Figure 2:**
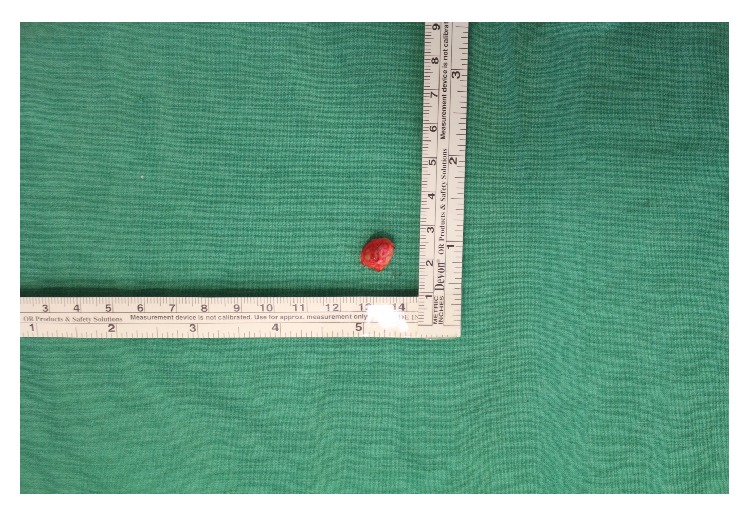
The gross appearance of the lesion indicates a unilocular cyst with brown mucinous contents.

A 7-year-old boy had a 3-year history of a subcutaneous cystic nodule on his left posterior neck area. The lesion was a solitary, painless, soft, and nontender subcutaneous nodule measuring approximately 1 cm in diameter (Figure 1). There was no history of previous trauma or remarkable medical problems. The mass had gradually increased in size but was otherwise asymptomatic. During surgical excision, the mass was revealed to be a cystic lesion located in the dermis and subcutaneous tissue. The cyst was subsequently excised with an overlying skin ellipse and sent for histological examination. The excised cyst was a unilocular cyst that was 1 cm at its greatest dimension (Figure 2).

**Figure 3:**
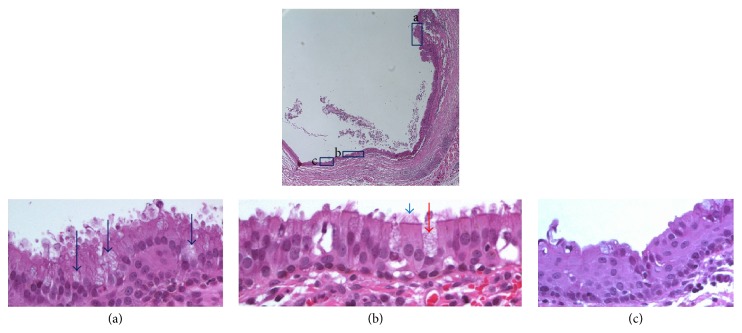
The cyst is covered by ciliated columnar epithelium with mucin vacuoles under low-power magnification (40x). (a) Mucin vacuoles are observed with ciliated columnar epithelium (dark blue arrows); hematoxylin and eosin (400x). (b) The inset shows cilia on both the top portion (blue arrow) and the lateral borders (red arrow) of the columnar cells (400x). (c) Squamous metaplasia (400x).

**Figure 4:**
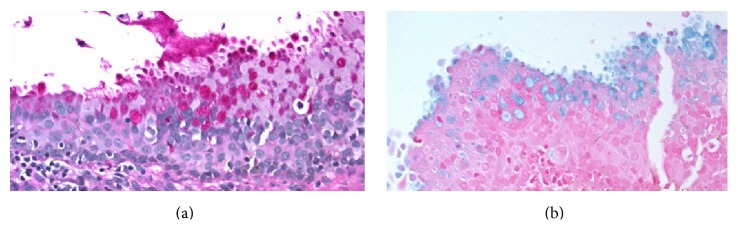
The mucin vacuoles are positive for the periodic acid-Schiff (PAS) and alcian blue stains. (a) Positivity to PAS. (b) Positivity to alcian blue (400x).

**Figure 5:**
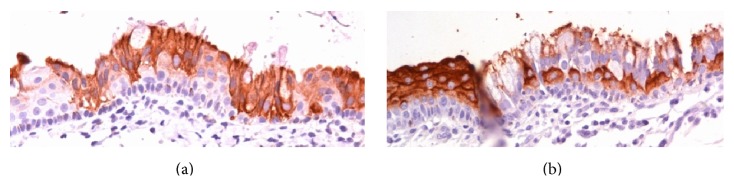
(a) The cytokeratin 7 staining is positive in columnar cells but negative for basal cells and squamous metaplastic cells (400x). (b) The immunohistochemical result of carcinoembryonic antigen (CEA) is the opposite (400x).

The specimen was stained with hematoxylin-eosin, alcian blue, and periodic acid-Schiff (PAS). Immunohistochemical studies were performed using antibodies to carcinoembryonic antigen (CEA), S-100 protein, ER, PR, epithelial membrane antigen (EMA), and cytokeratins 7 (CK 7) and 20 (CK 20). Microscopically, the cyst wall was lined by stratified columnar epithelia with mucin vacuoles and squamous metaplasia (Figure 3). Under high-power magnification, fine cilia were revealed on the luminal side of the epithelial lining and the lateral borders of the epithelia (Figures 3(a)–3(c)). The PAS and alcian blue stains were positive (Figure 4), and immunohistochemical staining revealed positivity to SMA, EMA, and CK 7 in the epithelial component. Immunoreactivities to p63, CK 20, S-100 protein, and ER and PR were negative. However, CEA was positive in the basal cells and squamous metaplasia and negative in the stratified columnar epithelium. The opposite result was observed for CK 7 staining (Figures 5(a) and 5(b)). Additionally, p63 staining was intensively positive in the squamous metaplasia.

## 3. Discussion

Cutaneous ciliated cysts are unusual benign lesions. In 1890, Hess first reported a case on the lower back of a 15-year-old girl. Farmer and Helwig later proposed the term cutaneous ciliated cyst for this entity after studying 11 cases in 1978 [2]. In their report, all 11 cases were observed on the lower extremities of female patients whose age ranged from 15 to 30 years, and the cyst wall was noted to have a ciliated epithelial lining resembling that of the fallopian tube [2]. The lesions typically measured several centimeters in diameter and presented as unilocular or multilocular cysts. A study describing the cysts histologically has reported that the cysts are typically lined by ciliated cuboidal to columnar epithelium with pseudostratified areas and that focal squamous metaplasia is occasionally present. Immunohistochemical studies have demonstrated that the cysts are PR and ER positive in all female cases, similar to the epithelia of the fallopian tubes [1].

Based on these findings, the Mullerian heterotopia hypothesis was proposed [1, 2]. The Mullerian heterotopia hypothesis suggests that cutaneous ciliated cysts arise as a sequestration of paramesonephric (Mullerian) duct structures during embryonic development [1–4, 13, 14]. From 6 to 7 weeks of gestation, the fallopian tubes develop from the unfused paramesonephric duct [15]. It is possible that these cells could detach and be incorporated within the lateral mesoderm and migrate locally in the area of the lower back, abdominal wall, or lower limb bud [1, 14, 15]. Mullerian rest cells arrest at various levels until hormonal stimulation at puberty, and then, following hormonal stimulation, the cells become functional, resulting in serous secretion and cyst formation [1, 14, 15]. Some authors support the Mullerian heterotopia hypothesis based on the following evidences [1, 2, 11, 14, 15].

1. Almost all cases arise in female patients.
2. Cutaneous ciliated cysts become apparent after puberty or during pregnancy.
3. Almost all of them are located on the lower limbs.
4. There is no histologic association with adnexal structures in female cases.
5. There are some reports of analogous noncutaneous lesions that are believed to have arisen from Mullerian rest cells.
6. The immunohistochemical staining profiles, including positive staining for ER and PR, are characteristic of the fallopian tube epithelium.
7. Ultrastructurally, the cilia show a 9 + 2 arrangement equal to normal human cilia.

However, outliers of cutaneous ciliated cysts, including those in male patients [5–11] and in unexpected locations such as the scalp [3], finger [16], and scapular area [17], have been reported. Thus, other hypotheses have been proposed including ciliated metaplasia of eccrine sweat glands [5, 8–11] and embryological remnants of the cloacal membrane [6, 7]. However, the cloacal membrane hypothesis was proposed simply because of the site of occurrence [6, 7].

There have been only 8 reported male patients in the 35 reports of cutaneous ciliated cysts. One case occurred on the scrotal skin of a 15-year-old male patient with a history of right cryptorchidism and orchiopexy [12]. Therefore, that case could be related to persistent Mullerian duct syndrome, a rare form of pseudohermaphroditism [1]. Excluding that case, there have been only 7 reported cases occurring in male patients. However, the pathologic findings differed between cases, including our case (Table 1). Three cases arose in the foot, two cases in the perineal area, and the other two cases in the cheek and inguinal area. In the last two cases, the authors reported negative staining for ER and PR and positive staining for PAS, EMA, and cytokeratin. However, the other results differed between the cases. Some authors have reported positive CEA staining [6, 11], but more cases have been reported to be negative. Based on these data, the eccrine metaplasia hypothesis was suggested. An eccrine origin is a possibility because fetal eccrine ducts [18] and eccrine spiradenoma [19] reportedly contain ciliated cells. However, normal eccrine glandular tissue stains positively for CEA [20], whereas most male cases were negative for CEA [5, 7–10] with the exception of only two cases [6, 11]. However, if the cutaneous ciliated cysts in male patients originated from Mullerian heterotopia, the cilia were degenerated in the microscopic examination because of prolonged estrogen depletion [21]. Some cases were represented by decapitation secretion, including our case, which is a typical feature of apocrine gland secretion [5, 9]. This may be additional evidence of eccrine metaplasia because many sweat gland lesions have both eccrine and apocrine differentiation within the same tumor [22].

Our case has some special characteristics compared to the other reported cutaneous ciliated cysts in male patients. The previous male cases were observed in patients from their late 20s to 60s, and no adolescent patients have been reported. Additionally, this is the first male case occurring on the shoulder area and the youngest case. Only one case in a female patient has been reported in the shoulder area [17]. However, the histopathological findings of our case are similar to most male cases including ciliated epithelia and negative CEA staining. However, focal squamous metaplasia was observed, and it was positive for CEA staining. Interestingly, the male cases positive for CEA were the oldest patients, with their ages being 56 and 60 years [6, 11]. It is possible that “delayed development” may lead to these results, but this is only a theory. All cases stained for SMA and PAS were positive, including our case. Based on these results, we propose that the cutaneous ciliated cysts in male patients originated from eccrine metaplasia.

## 4. Conclusion

Some authors have noted that the term cutaneous ciliated cyst is inaccurate and confusing, and they have suggested that cutaneous Mullerian cyst is more preferable [13]. As previously reported, we agree that cutaneous ciliated cysts should be divided into the subgroups of the cutaneous Mullerian cysts [13] and ciliated cutaneous eccrine cysts [16]. Cutaneous ciliated cysts presenting with positivity to ER and PR are classified as “cutaneous Mullerian cysts” and those that are negative should be classified as “ciliated cutaneous eccrine cysts.” To accurately evaluate the pathophysiology of ciliated cutaneous eccrine cysts, more cases need to be collected under exact criteria and compared with previously reported cases in various ways.

## Figures and Tables

**Table 1:** Summary of the reported cutaneous ciliated cysts in male patients. The empty columns mean that the results are not reported.

	Age	Site	PAS	SMA	Mucin	S100	CEA	EMA	ER/PR	CK	Others
Leonforte [5]	42	Lt. heel	+		−						
Trotter et al. [8]	28	Lt. foot			−		−	+		+	
Ashton [9]	25	Rt. sole		−	−	−	−	+		+	
Sidoni and Bucciarelli [6]	60	Perineal	+		+		+			+	Desmin+
Ohba et al. [10]	53	Rt. cheek	+	+		+	−	+	−	+	Desmin and vimentin−
Santos and Mendelsohn [7]	53	Perineal	+	−	+	−	−	+		+	Vimentin+
Lee et al. [11]	56	Rt. inguinal	+	+	−	−	+	+			
Pérez-Valcárcel et al. [12]	15	Scrotum	+						+		Cryptorchidism
Present case	7	Lt. shoulder	+	+	+	−	−	+	−	+	
